# Supplementary material for: Integrating full and partial genome sequences to decipher the global spread of canine rabies virus
Source: Nat Commun. 2023 Jul 17;14:4247. doi: 10.1038/s41467-023-39847-x (PMC10352342; doi:10.1038/s41467-023-39847-x)
Supplement: Supplementary file 1 — Supplementary Information [file 41467_2023_39847_MOESM1_ESM.pdf]

Supplementary Figures

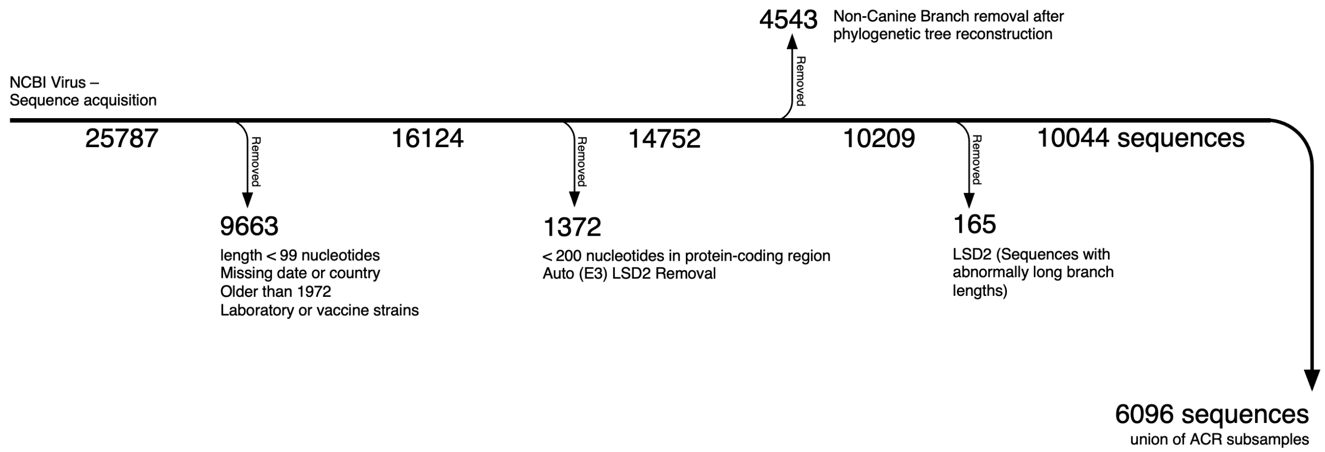

Supplementary Figure 1. Flowchart of inclusion and exclusion of RABV sequences downloaded from the NCBI Virus database. The number of sequences excluded and the reason for their removal is shown. The number of sequences present at each step is visible under the horizontal line.

RABV Gene Concatenation-Multiple Sequence Alignment Scheme

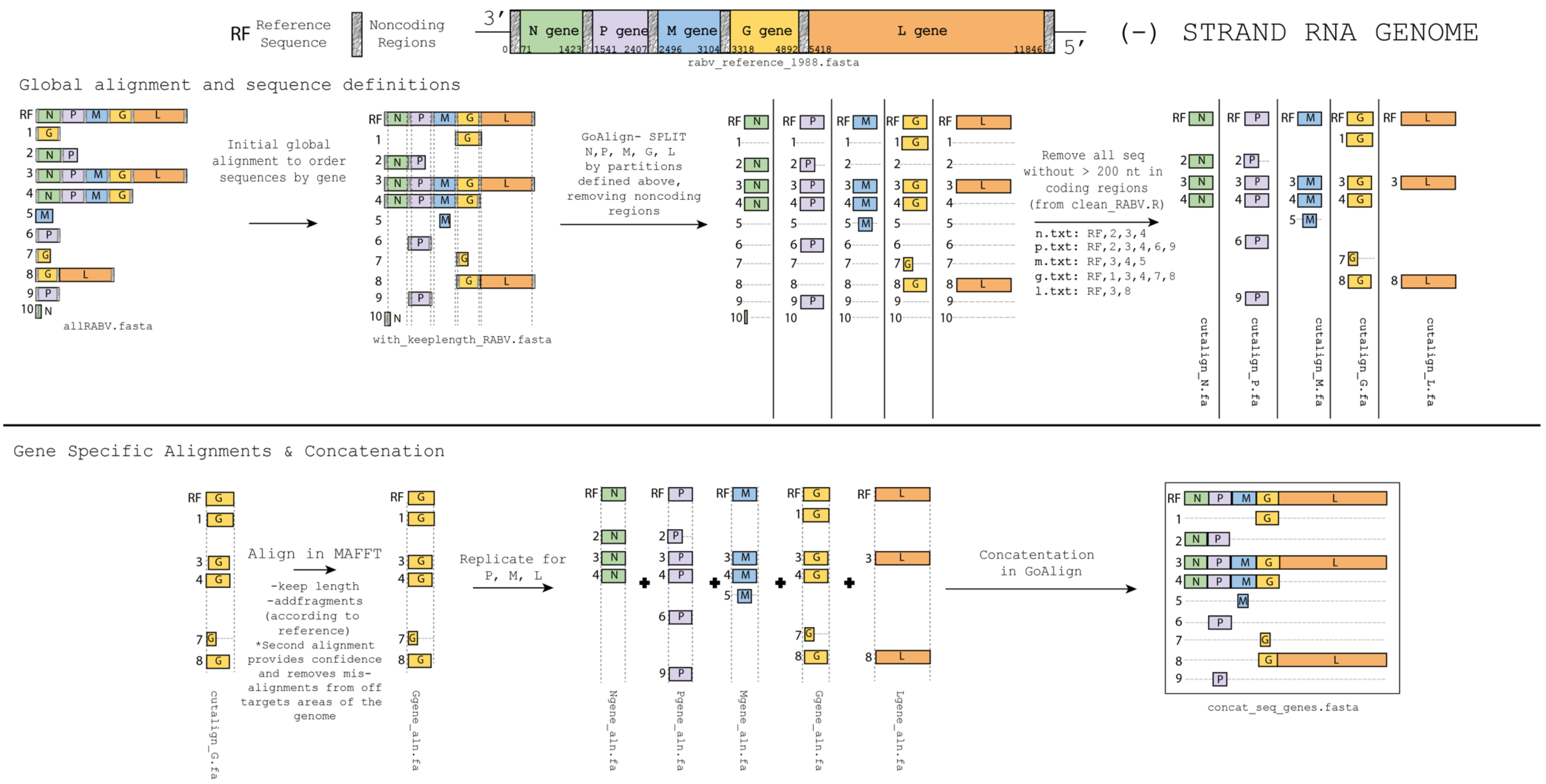

Supplementary Figure 2. Cartoon demonstration on the method of partial and WGS sequence concatenation. Sequences were defined by an initial global alignment using a custom script, and then sorted into gene-specific fasta files with sequences cut at the start codons of the corresponding gene. The gene-specific fasta files were aligned and concatenated to form a global multiple sequence alignment with a total length of 10,860 nucleotides. The illustration of the RABV genome is shown at the top. Numbers inside each gene area define the positions from the reference genome where the gene-specific sequences were cut. The final resulting multiple sequence alignment is shown in the box on the bottom right. Corresponding file names on the GitHub page (https://github.com/amholtz/GlobalRabies/tree/main/data/sequence_alignments/gene_specific_analysis) are given.

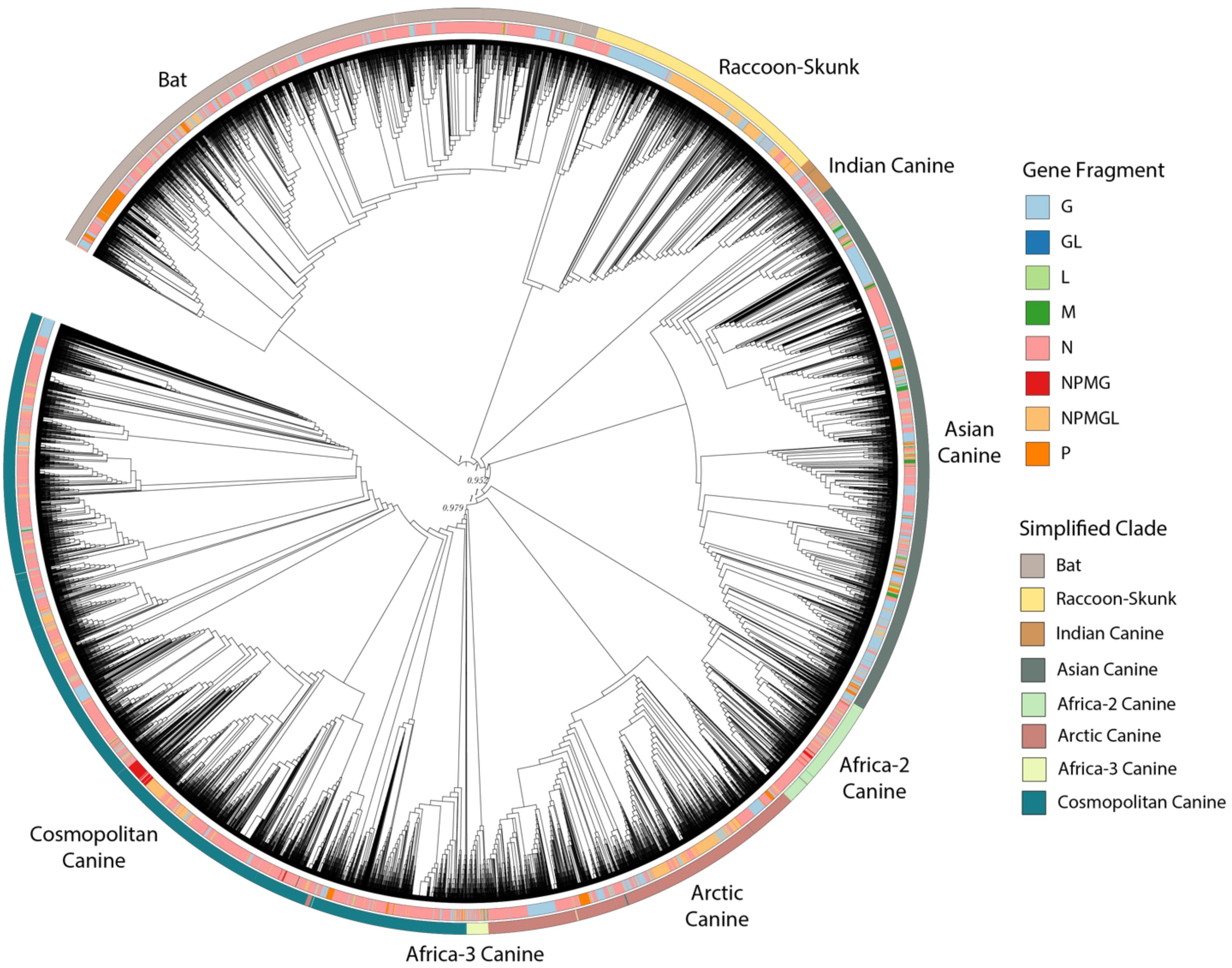

Supplementary Figure 3. Phylogenetic analysis of 14,752 RABV sequences. Sequences are labeled by simplified major clades (outer circle) and gene fragment (inner circle). Phylogenetic grouping by clade is clear, while there is a scattered distribution by gene fragment. Bootstrap support values (FastTrees Shimodaira-Hasegawa test) of simplified clade defining nodes are displayed.

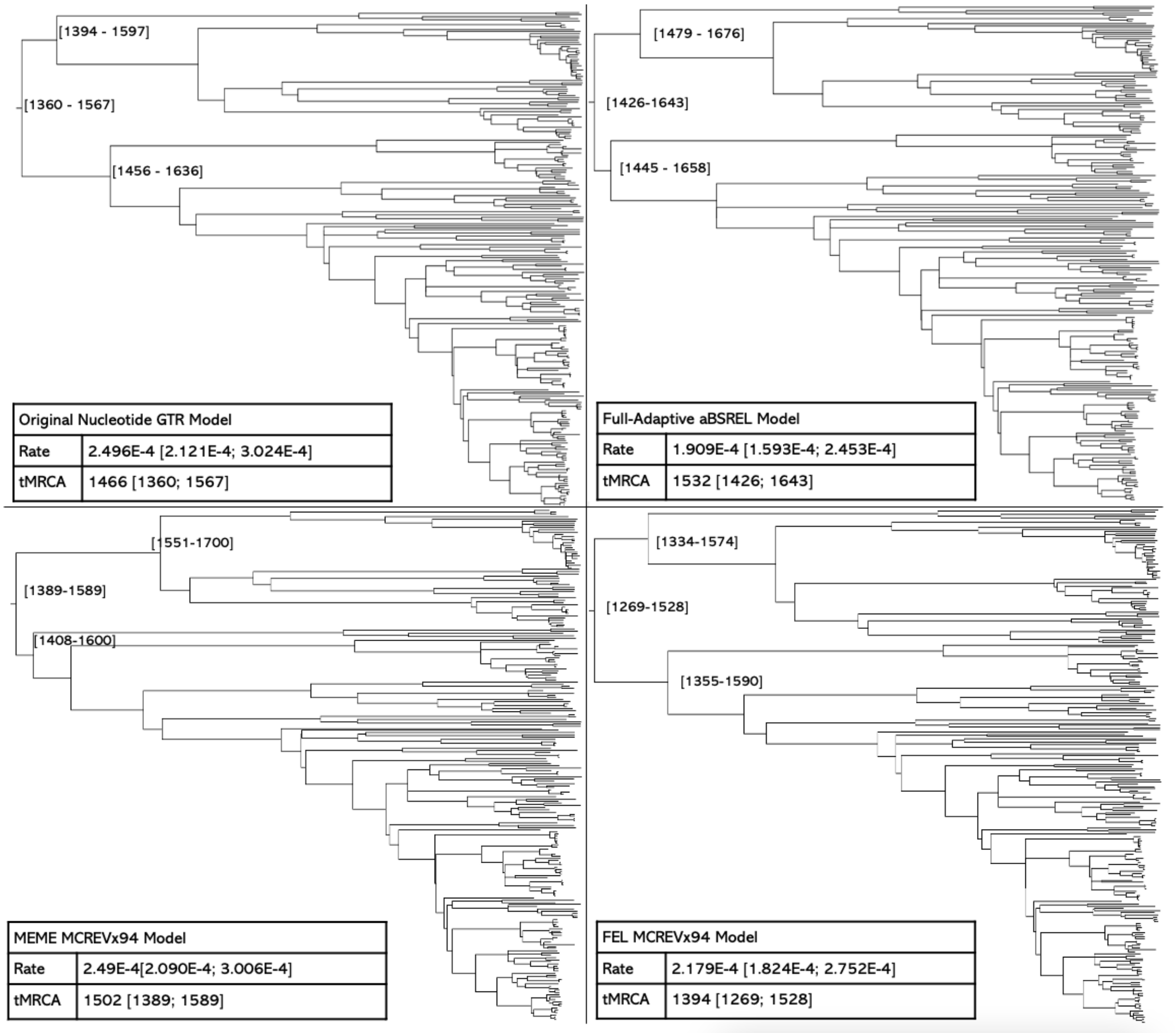

Supplementary Figure 4. Time-calibrated phylogenetic tree after branch-length optimization. Branch lengths were re-estimated by positive and purifying selection models in HyPhy by aBSREL, FEL, and MEME.

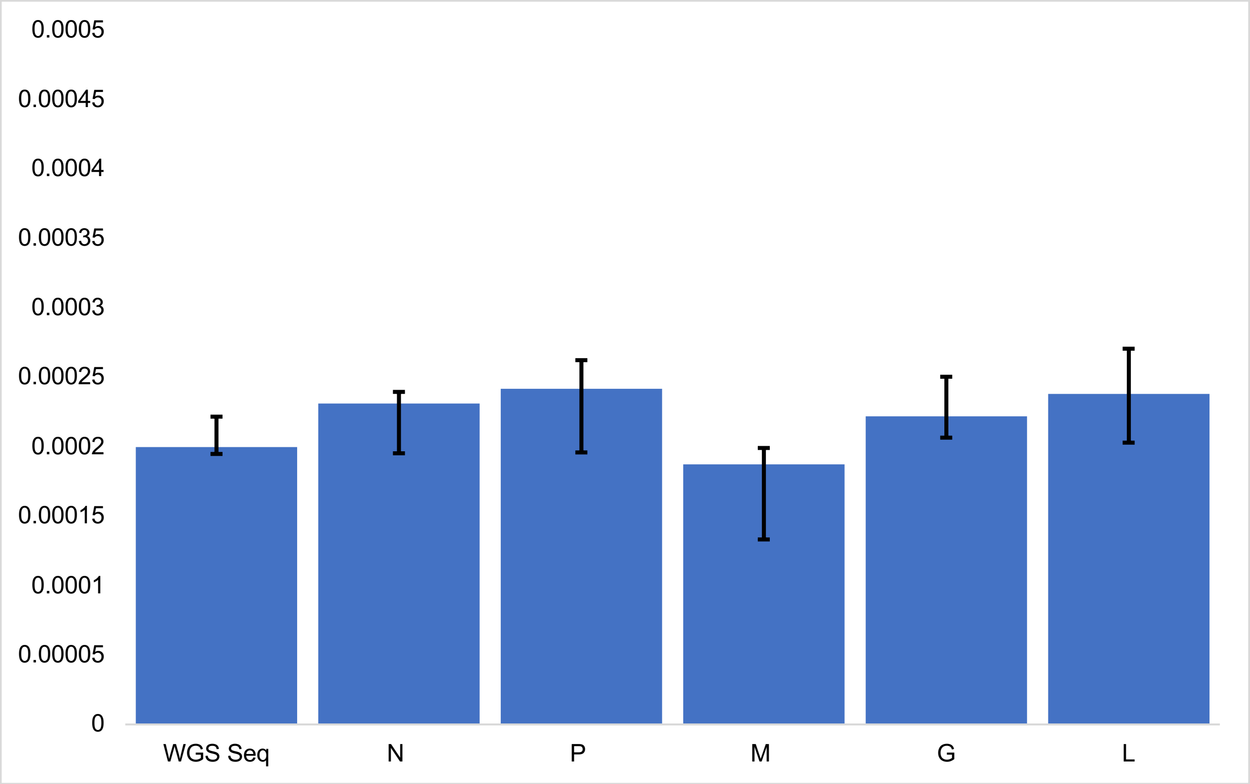

Supplementary Figure 5. Evolutionary rate of RABV genes in the canine-maintained cluster. Evolutionary rates were estimated by LSD2 from gene-specific MSAs with non-coding regions removed. Non-coding regions were not removed from WGS sequences. Regions tested include whole-genome sequences (WGS), nucleoprotein (N), phosphoprotein (P), matrix (M), glycoprotein (G) and polymerase (L). The point rate estimates are shown as vertical bars and are as follows: WGS (2.08E-4), N(2.17E-4), P(2.29E-4), M(1.66E-4), G(2.28E-4), L(2.37E-4). Error bars present 95% confidence intervals (CIs) from 1000 replicates. The LSD2 CIs are generated by parametric bootstrap: (i) A set of phylogenetic trees is generated by keeping the same topology as in the input tree and pooling each branch length from a Poisson distribution with the mean estimated by LSD2; (ii) The evolutionary rate is estimated on each simulated tree; (iii) The rate CIs are obtained from the 95% quantile of these rates.

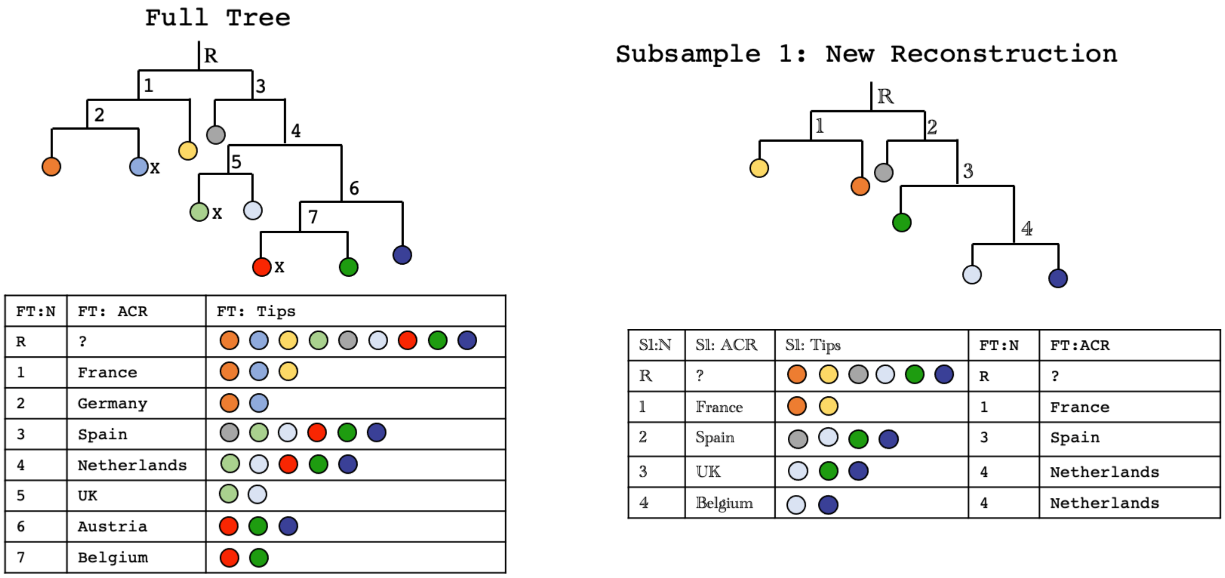

Supplementary Figure 6. Mock example of sub node comparison. Full Tree contains nine tips. Tips removed during subsampling are marked with an X. Subsample 1 contains 5 tips. Subsample 1 has been completely newly reconstructed using IQ-TREE2 starting from the subsampled sequence alignment. Notice that the topologies between the Full Tree (FT) and Subsample 1 are not identical (the light blue and green nodes have swapped). The goal is to find the node in Full Tree which represents the same tips in each node of Subsample 1. To do this, we want to find the node in Full Tree that contains all the tips found under a given node in Subsample 1. We pick the most specific node (i.e., with the least tips) among such nodes in the Full tree (or else each node in the subsample tree could be compared to the root since all tips are found under the root). In this example, node 4 in Subsample 1, can be compared to node 4 in Full Tree. Node 3 can be compared to node 4 as well. Node 2 in Subsample 1 can be compared to node 3 in Full Tree. Node 1 in Subsample 1 can be compared to Node 1 in Full Tree. If you now compare the ACR, you can see that the full tree nodes that share the same ACR are 1 and 3. This is the idea of the node comparison script which was applied on a much larger scale.

Supplementary Tables

Supplementary Table 1. Full RABV Metadata with added columns and exclusion reason
https://github.com/amholtz/GlobalRabies/blob/main/data/metadata_edited_exclusion.tab
https://amholtz.github.io/GlobalRabies/

Supplementary Table 2. Subgenomic fragments by sequence and country. Number of sequences and countries represented by each sequence fragment.

Fragment	Sequences	Countries
N	8136	120
G	3649	85
L	254	77
P	572	78
M	196	77
WGS	1972	75
Total	14779	121

Supplementary Table 3. Full Canine RABV tree compared to subsampled trees. Subsamples are the result of a subsampling protocol which produces a unique set of sequences for a more equal representation of countries. Internal nodes and their geographic estimates were compared to the full tree. Nodes with estimated character state probabilities greater than 50% were considered resolved. Internal nodes of two trees are compatible if their subtrees have the same tips. Of the compatible nodes in a subsample and the full tree, the estimated character (if resolved in both) was compared. 'Agg' column presents the full tree that has been pruned by the aggregation of sequences represented by subsamples 1-5. Triplet distance represents the fraction of triplets that differ across the trees normalized with all triplet possibilities in the compared trees.

	**Full Tree**	**Sub 1**	**Sub 2**	**Sub 3**	**Sub 4**	**Sub 5**	**Agg**
tMRCA	1356 [1301-1403]	1375 [1310- 1429]	1375 [1327-1416]	1369 [1310-1416]	1374 [1317-1423]	1368 [1311-1417]	1356 [1301-1403]
num. tips	10044	5371	5367	5367	5372	5371	6096
num. internal nodes	4779	2729	2724	2751	2713	2715	3114
normalized triplet distance to Full Tree	-	0.0062	0.0064	0.0062	0.0063	0.0060	-
# reso. internal nodes (%)	4706 (98.5%)	2673 (97.9%)	2665 (97.8%)	2687 (97.7%)	2646 (97.5%)	2656 (97.8%)	-
inter. nodes Full Tree compatible (%)	-	60.16%	60.84%	59.03%	59.94%	59.65%	-
Full Tree shared ACR (of compatible)	-	1770 (94.40%)	1741 (94.47%)	1817 (94.24%)	1778 (94.32%)	1787 (94.10%)	2169 (93.61%)

Supplementary Table 4. Ancestral character reconstruction (ACR) by region and country of both parental and child node for 44 canine subclades. 95% confidence intervals are provided for the tMRCA for each clade. tMRCA of previous Troupin et al. [26] are displayed. Rows with more than one country show the conflict between the confidence analysis between the full tree ACR result and the subsamples (Full Tree | Subsampled aggregation).

Clade Definition	Regional Origin	Country Origin	TMCRA	Troupin et al. [26] TMRCA	Parent Region	Parent Country	Parent - TMRCA
Africa-2	Western Africa		1799 (1761 - 1832)	1802 (1750 - 1852)			1578 (1522 - 163)
Africa-3	Southern Africa	South Africa	1723 (1691 - 1752)	1756 (1710 - 1815)			1564 (1526 - 159)
Arctic_A	Northern America	Canada	1929 (1918 - 1939)	1942 (1929 - 1954)			1796 (1766 - 1824)
Arctic_AL1a	Southern Asia		1920 (1905 - 1931)	1940 (1927 - 1953)	Southern Asia		1879 (1859 - 1898)
Arctic_AL1b	Southern Asia		1948 (1940 - 1954)	1936 (1919 - 1953)	Southern Asia		1938 (1931 - 1945)
Arctic_AL2	Eastern Asia	China	1866 (1843 - 1885)	1886 (1852 - 1921)			1743 (1709 - 1777)
Arctic_AL3	Southern Asia	Nepal	1977 (1973 - 1981)	1881 (1856 - 1906)	Southern Asia		1907 (1892 - 1921)
Asian_SEA1a	Eastern Asia	China	1904 (1880 - 1926)	1973 (1967 - 1978)	Eastern Asia		1711 (1675 - 1747)
Asian_SEA1b	Eastern Asia		1768 (1737 - 1797)	1830 (1789 - 1873)	Eastern Asia		1711 (1675 - 1747)
Asian_SEA2a	Eastern Asia	China	1826 (1799 - 1850)	1956 (1945 - 1967)	Eastern Asia		1740 (1701 - 1773)
Asian_SEA2b	Eastern Asia	China	1975 (1966 - 1982)	1951 (1937 - 1968)	Eastern Asia	China	1857 (1831 - 1881)
Asian_SEA3	South-Eastern Asia	Thailand	1853 (1827 - 1876)	1898 (1876 - 1920)	Eastern Asia		1589 (1553 - 1623)
Asian_SEA4	South-Eastern Asia	Philippines	1898 (1885 - 1910)	1925 (1904 - 1946)	Eastern Asia		1671 (1635 - 1701)
Asian_SEA5	Eastern Asia	Taiwan	1791 (1762 - 1819)	1957 (1939 - 1973)	Eastern Asia		1651 (1616 - 1680)
Cosmopolitan	Northern America		1656 (1627 - 1683)	1730 (1687 - 1773)			1564 1526 - 1598)
Cosmo_AF1a	Northern Africa		1876 (1862 - 1889)	1872 (1851 - 1895)	Northern Africa		1854 (1842 - 1866)
Cosmo_AF1b	Eastern Africa	Zambia	1878 (1863 - 1891)	1907 (1890 - 1925)	Eastern Africa		1826 (1813 - 1838)
Cosmo_AF1c	Eastern Africa	Madagascar	1983 (1980 - 1985)	1983 (1980 - 1985)	Eastern Africa		1826 (1813 - 1838)
Cosmo_AF4	Northern Africa	Egypt	1910 (1894 - 1927)	1932 (1923 - 1940)	Northern America	USA	1679 (1656 - 1701)
Cosmo_AM1	Northern America	USA	1867 (1850 - 1883)	1908 (1886 - 1928)	Northern America	USA	1810 (1794 - 1825)
Cosmo_AM2a	Central America	Mexico	1851 (1836 - 1864)	1890 (1861 - 1920)	Northern America	USA	1772 (1750 - 1791)
Cosmo_AM2b	Northern America	USA	1939 (1928 - 1949)	1830 (1781 - 1852)	Northern America	USA	1791 (1768 - 1811)
Cosmo_AM3a	South America	Brazil	1897 (1880 - 1912)	1912 (1890 - 1936)	South America	Brazil	1808 (1785 - 1827)
Cosmo_AM3b	South America	Brazil	1861 (1847 - 1872)	1890 (1863 - 1916)	South America	Brazil	1808 (1785 - 1827)
Cosmo_AM4	Northern America	USA	1821 (1800 - 1840)	1846 (1811 - 1883)	Northern America	USA	1710 (1688 - 1732)
Cosmo_CA1	Eastern Europe	Russia	1924 (1913 - 1935)	1946 (1936 - 1957)	Eastern Europe		1898 (1889 - 1908)
Cosmo_CA2	Western Asia	Iraq	1981 (1977 - 1985)	1944 (1927 - 1960)	Western Asia		1817 (1798 - 1833)
Cosmo_CA3	Eastern Europe	Romania| Russia	1936 (1925 - 1946)	1942 (1926 - 1956)	Eastern Europe		1880 (1870 - 1889)
Cosmo_CE	Eastern Europe	Germany| Poland	1968 (1962 - 1973)	1967 (1961 - 1974)	Eastern Europe	Germany| Poland	1931 (1921 - 1938)
Cosmo_EE	Southern Europe	Serbia	1944 (1936 - 1950)	1943 (1934 - 1954)	Eastern Europe	Poland|Serbia	1907 (1897 - 1916)
Cosmo_ME1a	Western Asia		1922 (1911 - 1933)	1938 (1927 - 1948)	Western Asia		1861 (1850 - 1872)
Cosmo_ME1b	Western Asia	Israel	1990 (1987 - 1991)	1987 (1984 - 1990)	Western Asia		1922 (1911 - 1933)

Cosmo_ME2	Western Asia	Turkey	1960 (1952 - 1968)	1986 (1984 - 1989)	Western Asia		1859 (1847 - 1870)
Cosmo_NEE	Eastern Europe		1887 (1873 - 1899)	1954 (1926 - 1964)	Eastern Europe	Poland|Serbia	1882 (1870 - 1892)
Cosmo_Vac	Western Europe	France	1959 (1950 - 1967)	not in study	Northern America	USA	1788 (1771 - 1805)
Cosmo_Vac2	Central America	Mexico	1942 (1926 - 1954)	not in study	Northern America	USA	1864 (1845 - 1880)
Cosmo_WE	Western Europe	Germany	1948 (1940 - 1954)	1949 (1942 - 1958)	Eastern Europe	Germany| Poland	1931 (1921 - 1938)
Cosmo_YUGCOW	Southern Europe	Montenegro	1977 (1977 - 1979)	not in study	Western Asia		1819 (1804 - 1833)
Cosmo_YUGFOX	Southern Europe	Serbia	1967 (1954 - 1977)	not in study	Southern Europe	Serbia	1909 (1894 - 1923)
Indian-Sub	Southern Asia		1760 (1716 - 1799)	1785 (1733 - 1840)			1397 (1341 - 1443)

Supplementary Table 5. Inferred human-mediated introductions identified on the phylogenetic tree. Using the full-canine tree consisting of 10,044 sequences, 14,640 transmissions were identified, 232 of which were to non-neighboring countries, and 43 of which fit the criteria as a human-mediated introduction. Human-mediated introductions are defined as transmissions that occurred within 16 years and between two countries that are more than 2000 km apart or separated by a body of water.

Parent Country	Child Country	Parent Date	Child Date	Branch Length (years)	Subtree Size (num. of tips)	Distance (km)	(km/year)
Cameroon	Tanzania	1992.8	1993.0	0.2	73.0	3308.4	19477.4
China	Chile	1996.4	1998.0	1.6	1.0	19079.9	11754.8
Bangladesh	France	2014.6	2016.5	1.9	1.0	7916.8	4131.3
India	Oman	2008.5	2009.0	0.5	1.0	1936.2	3556.2
India	Oman	2007.9	2008.7	0.8	13.0	1936.2	2442.4
Philippines	Japan	2004.5	2006.0	1.5	1.0	2999.5	2003.3
Philippines	Japan	2004.5	2006.0	1.5	1.0	2999.5	2003.3
Philippines	Japan	2004.1	2006.0	1.9	1.0	2999.5	1605.9
India	Nigeria	2002.0	2007.7	5.7	3.0	8088.0	1430.3
France	India	1994.0	1999.0	5.0	5.0	6594.2	1318.8
India	France	2009.0	2014.0	5.0	1.0	6594.2	1316.7
France	Mexico	1994.0	2002.5	8.5	3.0	9206.8	1079.8
Nigeria	Iran	2007.7	2013.2	5.5	1.0	5865.2	1065.1
Nepal	Italy	1990.0	1997.0	7.0	1.0	6640.5	948.6
India	Italy	2004.7	2011.9	7.1	1.0	5922.2	831.8
Mali	France	2009.0	2014.2	5.3	1.0	4139.8	785.9
Thailand	Philippines	2001.0	2004.0	3.0	1.0	2210.0	736.0
India	Ethiopia	2000.9	2008.0	7.1	1.0	4561.5	644.2
Thailand	Philippines	2001.0	2005.0	4.0	1.0	2210.0	552.5
China	Thailand	2005.0	2012.0	7.0	1.0	3303.9	471.8
Nepal	Italy	1981.3	1996.0	14.7	1.0	6640.5	452.2
Namibia	Tanzania	2004.0	2011.0	7.0	1.0	2953.5	421.9
China	Thailand	2005.0	2013.0	8.0	1.0	3303.9	412.8
China	Thailand	2005.0	2013.0	8.0	1.0	3303.9	412.8
China	Thailand	2005.0	2013.0	8.0	1.0	3303.9	412.8
China	Thailand	2005.0	2013.0	8.0	1.0	3303.9	412.8
China	Thailand	2005.0	2013.0	8.0	1.0	3303.9	412.8
China	Thailand	2005.0	2013.0	8.0	1.0	3303.9	412.8
China	Thailand	2005.0	2013.0	8.0	1.0	3303.9	412.8
Tanzania	Cameroon	1993.0	2001.5	8.5	24.0	3308.4	388.3
China	Thailand	2005.0	2014.0	9.0	1.0	3303.9	367.0
China	Thailand	2005.0	2014.0	9.0	1.0	3303.9	367.0

Tanzania	Ghana	1995.9	2008.7	12.9	1.0	4604.6	357.4
India	Thailand	2009.0	2017.9	8.9	1.0	2919.7	327.1
India	Iran	1999.0	2007.5	8.5	2.0	2544.6	297.6
China	Thailand	2002.5	2014.0	11.5	2.0	3303.9	286.3
Tanzania	Cameroon	1993.0	2006.5	13.5	3.0	3308.4	244.5
Mauritania	Nigeria	1981.5	1993.0	11.5	1.0	2478.3	216.1
Cuba	Colombia	1905.6	1917.5	12.0	3.0	2203.7	184.4
Mexico	Cuba	1894.2	1905.6	11.3	109.0	1784.2	157.7
Morocco	Spain	1958.9	1973.4	14.5	9.0	762.7	52.8
Spain	Morocco	1992.5	2008.0	15.5	1.0	762.7	49.3

Supplementary Methods

Supplementary Methods 1. Purifying Selection Models used from HyPhy
FEL identifies instances of purifying selection in codons and identifies the internal branches where this has occurred. MEME identifies instances of episodic and pervasive positive selection on internal branches of the tree. aBSREL uses an adaptive model to re-estimate dN/dS ratios for each branch of the tree, yielding a more specific branch-length estimation.

Supplementary Methods 2. ACR Comparison Between Full-Canine Tree and Subsamples
A custom script (https://github.com/amholtz/GlobalRabies/blob/main/R/ACR_Sub_comparison.R) was used to compare the ACR node estimations for each subsample and full-canine tree (see Supp. Figure 6). We considered nodes with ACR marginal probability > 50% as "resolved" and the others as "unresolved". 98% of the tree nodes were resolved in the full-canine tree and in the subsampled trees (see Supp. Table 3 for more details). The state estimates were aggregated across each subsample and compared to the full-canine tree PastML node estimates. Only state (country) estimates consistent between the aggregated subsample and full-canine tree were retained. Of 4706 nodes that had resolved ancestral state (country) estimates from the full-tree, 1103 were found across the 5 subsamples (23%) and could hence be compared. Most nodes that were not comparable were peripheral on the tree. A total of 90% of the comparable nodes share the same ancestral state estimates as the full-canine tree estimate. This result strongly validates the original ancestral character reconstruction (ACR) for the full-canine tree, indicating how only about 10% of the nodes alter after subsampling.
