# Supplementary material for: Integrating full and partial genome sequences to decipher the global spread of canine rabies virus
Source: Nat Commun. 2023 Jul 17;14:4247. doi: 10.1038/s41467-023-39847-x (PMC10352342; doi:10.1038/s41467-023-39847-x)
Supplement: Supplementary file 3 — Reporting Summary [file 41467_2023_39847_MOESM3_ESM.pdf]

nature portfolio

Corresponding author(s): Andrew Holtz and Anna Zhukova

Last updated by author(s): 06/21/2023

Reporting Summary

Nature Portfolio wishes to improve the reproducibility of the work that we publish. This form provides structure for consistency and transparency in reporting. For further information on Nature Portfolio policies, see our Editorial Policies and the Editorial Policy Checklist.

Statistics

For all statistical analyses, confirm that the following items are present in the figure legend, table legend, main text, or Methods section.

n/a	Confirmed	
☐	☒	The exact sample size (*n*) for each experimental group/condition, given as a discrete number and unit of measurement
☒	☐	A statement on whether measurements were taken from distinct samples or whether the same sample was measured repeatedly
☐	☒	The statistical test(s) used AND whether they are one- or two-sided<br>*Only common tests should be described solely by name; describe more complex techniques in the Methods section.*
☐	☒	A description of all covariates tested
☐	☒	A description of any assumptions or corrections, such as tests of normality and adjustment for multiple comparisons
☒	☐	A full description of the statistical parameters including central tendency (e.g. means) or other basic estimates (e.g. regression coefficient) AND variation (e.g. standard deviation) or associated estimates of uncertainty (e.g. confidence intervals)
☐	☒	For null hypothesis testing, the test statistic (e.g. *F*, *t*, *r*) with confidence intervals, effect sizes, degrees of freedom and *P* value noted<br>*Give P values as exact values whenever suitable.*
☒	☐	For Bayesian analysis, information on the choice of priors and Markov chain Monte Carlo settings
☐	☒	For hierarchical and complex designs, identification of the appropriate level for tests and full reporting of outcomes
☒	☐	Estimates of effect sizes (e.g. Cohen's *d*, Pearson's *r*), indicating how they were calculated

Our web collection on statistics for biologists contains articles on many of the points above.

Software and code

Policy information about availability of computer code

Data collection	Data was collected using NCBI Virus Database. More information is given below under 'Data'.
Data analysis	Data was analysed using softwares: MAFFT(v7.505), Python(v3.8.5), R(v4.2.1), FastTree(v2.1.11), Goalign(v0.3.5), Gotree(v0.4.4), TempEst(v1.5.3), HyPhy(v2.5.40), IQTREE2(v2.2.2.2), LSD2(v1.8.8), PastML(v.1.9.34), and iTol (v6.7.5). More information can be found on our github (https://github.com/amholtz/GlobalRabies)

For manuscripts utilizing custom algorithms or software that are central to the research but not yet described in published literature, software must be made available to editors and reviewers. We strongly encourage code deposition in a community repository (e.g. GitHub). See the Nature Portfolio guidelines for submitting code & software for further information.

Data

Policy information about availability of data

All manuscripts must include a data availability statement. This statement should provide the following information, where applicable:

- Accession codes, unique identifiers, or web links for publicly available datasets
- A description of any restrictions on data availability
- For clinical datasets or third party data, please ensure that the statement adheres to our policy

Data was downloaded via NCBI Virus (https://www.ncbi.nlm.nih.gov/labs/virus/vssi/#/virus?SeqType_s=Nucleotide&VirusLineage_ss=Lyssavirus%20rabies,%20taxid:11292). Information on geography, country codes, colonization was incorporated via R packages: cepiigeogist, countrycode. More information can be found

on our github (https://github.com/amholtz/GlobalRabies). The datasets generated during and/or analysed during the current study are available in the github repository, https://github.com/amholtz/GlobalRabies. Permanent Zenodo link: DOI: 10.5281/zenodo.8047854

Human research participants

Policy information about studies involving human research participants and Sex and Gender in Research.

Reporting on sex and gender	not applicable
Population characteristics	not applicable
Recruitment	not applicable
Ethics oversight	not applicable

Note that full information on the approval of the study protocol must also be provided in the manuscript.

Field-specific reporting

Please select the one below that is the best fit for your research. If you are not sure, read the appropriate sections before making your selection.

☒ Life sciences ☐ Behavioural & social sciences ☐ Ecological, evolutionary & environmental sciences

For a reference copy of the document with all sections, see nature.com/documents/nr-reporting-summary-flat.pdf

Life sciences study design

All studies must disclose on these points even when the disclosure is negative.

Sample size	Sampling size was determined by the number of rabies virus sequences available for download on NCBI Virus. The sample size for the canine subsection was determined simply by pruning the original tree for all sequences clustering under the canine branch. The size for the canine tree subsamples for IQTREE estimation was determined by weighing time requirements for tree reconstruction against the number of country locations we wanted to include in the study. This size is sufficient for analysis since the topology of the trees matched the topology of the original tree without subsampling.
Data exclusions	A quality check was conducted to remove sequences that were:<br>(1) missing date and country information - questions data integrity and this information is important for our study<br>(2) older than 1972 - questionable and error prone sequencing methods prior to 1972<br>(3) identified as vaccine or laboratory strains - vaccine and laboratory strains replicate in the lab and do not provide relevant phylogeographic information<br>(4) with coding regions shorter than 200 nucleotides - shorter than 200 nucleotides could lead to nonspecific alignment to other parts of the genome. In addition, the short sequence could be the result of sequencing error.
Replication	Tree reconstruction via FastTree was verified by 5500 sequence subsamples trees, reconstructed via IQTREE2 with gene partitioning. All subsamples constructed via IQTREE2 with gene partitioning verify the phylogenetic estimations by FastTree (triplet distance testing) and by dating estimations by LSD2.
Randomization	Samples were grouping into three categories:<br>(1) Clade - sequences were allocated into clade groupings according to where they cluster on the phylogenetic tree. This was determined previously using the tool RABV Glue (http://rabv-glue.cvr.gla.ac.uk/)<br>(2) Regions - sequences were allocated into regional groupings by World Bank Regional Definitions according to the R package cepiigeogist<br>(3) Colonial Empire - sequences were allocated into colonial history groups by their colonial past (French, British, Spanish, Russian, Portugese, and Noncolonial).
Blinding	Blinding was not relevant to this study, since phylogeographic studies rely on objective genetic data, minimizing the influence of human bias or interventions. Sampling bias, however, is a consideration in phylogeography studies, and we addressed this by implementing subsampling techniques to ensure representative data collection.

Reporting for specific materials, systems and methods

We require information from authors about some types of materials, experimental systems and methods used in many studies. Here, indicate whether each material, system or method listed is relevant to your study. If you are not sure if a list item applies to your research, read the appropriate section before selecting a response.

Materials & experimental systems

n/a	Involved in the study
☒	☐ Antibodies
☒	☐ Eukaryotic cell lines
☒	☐ Palaeontology and archaeology
☒	☐ Animals and other organisms
☒	☐ Clinical data
☒	☐ Dual use research of concern

Methods

n/a	Involved in the study
☒	☐ ChIP-seq
☒	☐ Flow cytometry
☒	☐ MRI-based neuroimaging
